# Mental disorders in pregnancy and 5–8 years after delivery

**DOI:** 10.1017/gmh.2016.26

**Published:** 2016-11-23

**Authors:** P. H. C. Rondó, R. F. Ferreira, J. O. Lemos, J. A. Pereira-Freire

**Affiliations:** 1 Department of Nutrition, School of Public Health, University of São Paulo, Avenida Dr Arnaldo 715, São Paulo, CEP: 01246-904, Brazil; 2 Federal University of Piauí, Campus Senador Helvídio Nunes de Barros, Rua Cícero Eduardo s/n, Bairro Junco, Picos PI, CEP: 64600-000, Brazil

**Keywords:** Anxiety, maternal depression, postpartum, pregnancy, stress

## Abstract

**Background.:**

Even though mental disorders represent a major public health problem for women and respective children, there remains a lack of epidemiological longitudinal studies to assess the psychological status of women throughout pregnancy and later in life. This epidemiological cohort study assessed the relationship between mental disorders of 409 Brazilian women in pregnancy and 5–8 years after delivery.

**Methods.:**

The women were followed from 1997 to 2000 at 17 health services, and subsequently from 2004 to 2006 at their homes. Mental disorders were investigated by the Perceived Stress Scale-PSS, General Health Questionnaire-GHQ and State-Trait Anxiety Inventories-STAI. The relationship between scores of the PSS, GHQ and STAI 5–8 years after delivery and in pregnancy was assessed by multivariate linear regression analysis, controlling for the following confounders: maternal age, education, *per capita* income, family size, work, marital status and body mass index.

**Results.:**

Scores of the PSS, GHQ and STAI 5–8 years after delivery were positively associated with scores of the PSS, GHQ and STAI in the three trimesters of pregnancy, and inversely associated with maternal age and *per capita* income (adj. *R*^2^ varied from 0.15 to 0.37). PSS, GHQ and STAI scores in the 3rd trimester of pregnancy were positively associated with scores of the PSS, GHQ and STAI in the 1st and 2nd trimesters of pregnancy (adj. *R*^2^ varied from 0.31 to 0.65).

**Conclusions.:**

The results of this study reinforce the urgency to integrate mental health screening into routine primary care for pregnant and postpartum women.

## Introduction

According to the WHO World Mental Health (WMH) Surveys (Kessler *et al.* 2015) involving 24 countries, approximately half of the individuals with major depressive disorder (MDD) had previously anxiety disorders. Women and formerly married people were the groups most at risk for elevated rates of MDD associated with anxiety disorders.

It is well recognized that during women's life pregnancy and postpartum are very sensitive periods, predisposing them to mental disorders like anxiety and depression (Howard *et al*. 2014). Mental disorders in pregnancy have been associated with prematurity and low birth weight (Rondó *et al.* 2003; Rondó, 2007; Grote *et al.* 2010). When present in the postpartum period, these disorders can have a negative impact on the mental health of the children (Buss *et al.* 2011; Davis & Sandman, 2012; Loomans *et al.* 2012; Ali *et al.* 2013; Rouse & Goodman, 2014), and even a few years later in life, they may interfere in the nutritional status of the children (Dunkel Schetter, 2011; Ali *et al.* 2013; Rondó *et al.* 2013).

Mental disorders, if firstly observed in pregnancy and in the postpartum periods, may be transitional, but can also persist throughout life (Kim *et al.* 2008; Mora *et al.* 2009; Sutter-Dallay *et al.* 2012; Kuo *et al.* 2014), bringing several problems for the women and the whole family.

Even though mental disorders represent a major public health problem for women and respective children, there remains a lack of epidemiological longitudinal studies to assess the psychological status of women throughout pregnancy and later in life. Most studies emphasized the pregnancy and/or the immediate post-partum periods (Haim *et al.* 2014; Radoš *et al.* 2015), but did not follow women years after delivery.

Therefore, the objective of this study was to follow women throughout pregnancy up to 5–8 years after delivery to assess the relationship between mental disorders in the three trimesters of pregnancy and later in life.

## Methods

This epidemiological cohort study involved 409 women in Jundiai city, São Paulo state, Brazil. It is derived from a cohort, carried out between 1997 and 2000, initially composed of 865 low-income pregnant women from Jundiai, to evaluate stress and distress as predictors of low birth weight, prematurity and intrauterine growth retardation. The women were recruited from all health units and hospitals in the city and were followed before the 16th week of pregnancy to the birth of their babies. All women were insured by the National Health Service (SUS) and were apparently healthy, considering that those who present any problem in pregnancy are usually reported to specialized antenatal services. Women with chronic infectious diseases, metabolic diseases, cardiopathy, mental diseases, hypertension/pre-eclampsia/eclampsia, vaginal bleeding and multiple deliveries were excluded from the study. Details of the cohort have been published previously (Rondó *et al.* 2003; Rondó & Souza, 2007).

The second prospective cohort study was carried out between November 2004 and December 2006 to assess mental disorders 5–8 years after delivery, and consisted of two phases. In the first phase, information from the questionnaire of the first cohort study was taken into account, and the women who at that time were living in Jundiai and nearby municipalities were located. The women were invited to participate in the present study through telephone contact or by visiting their homes if they did not have a telephone. Next, a home visit was made, during which the objective of the study was explained, and the ethical consent form was signed by the women. At the time of this home visit, a general questionnaire was applied in order to assess demographic and socioeconomic factors. In the second phase of the study, the participants were contacted again by telephone to arrange to collect anthropometric measurements and data on mental disorders.

Out of the 865 women from the previous cohort, 745 women were located and invited to participate in the study, resulting in a sample of 649 women who signed a free informed consent form and answered a general questionnaire. However, 240 of them had incomplete data or did not participate in the second phase of the study, resulting in a final sample of 409 women.

Demographic and socioeconomic characteristics of the women were evaluated by the same general questionnaire used in the original cohort study. Their nutritional status was determined by the body mass index (BMI) and classified according to WHO recommendations (WHO, 2000). The women were weighed on a portable Sohnle^®^ electronic scale (Sohnle, model 7500, Murrhardt, Germany), with a precision of 100 g. Height was measured with a SECA^®^ wall-mounted stadiometer (Leicester Portable Height measure model, Hamburg, Germany), with a precision of 0.1 cm. The anthropometric measurements were performed according to the recommendations of Jelliffe & Jelliffe (1989).

Mental disorders were assessed by four psychologists who interviewed the women 3 times in pregnancy (at a gestational age <16 weeks, from 20 to 26 weeks and from 30 to 36 weeks) and 5–8 years after delivery, using versions of the Perceived Stress Scale (PSS), the General Health Questionnaire (GHQ) and the State-Trait Anxiety Inventories (STAI) validated in Brazil, respectively, by Luft *et al.* (2007), Mari & Williams (1985) and Biaggio *et al.* (1977). The PSS (Cohen *et al.* 1983) determined the degree to which situations in the last month had been considered as stressful, on a five-point scale ranging from ‘never’ to ‘very often’. A 12-item version of the GHQ (Mari & Williams, 1985) assessed mental disorders in general, and classified the individuals in two groups: with low, 0–3 and high, ⩾4 scores. The STAI (Spielberger *et al.* 1970) assessed anxiety by a well standardized, 40 item, self-report instrument designed to measure both state and trait anxiety. For State Anxiety (SA), subjects were asked how they felt at the time of being questioned, and for Trait Anxiety (TA), subjects were asked how they felt generally. A cut-off point greater than 40 was selected for both SAI and TAI.

The Stata version 10 software (College Station, TX, USA) was used for storage and statistical analysis of the data. The relationship between scores of the PSS, GHQ and STAI 5–8 years postpartum (dependent variables) and in the three trimesters of pregnancy (independent variables) was assessed by multivariate linear regression analysis, using the backward stepwise selection method. The relationship between scores of the PSS, GHQ and STAI in the 3rd trimester of pregnancy (dependent variables) and in the 1st and 2nd trimesters (independent variables) was also assessed. The following confounding factors were included in the models: maternal age, education, *per capita* income, family size, work, marital status and BMI. A *p* value ⩽0.05 was considered as statistically significant.

## Results

There were losses of 33.6% and 42.7% of the sample, respectively, considering all the mothers who were located (*n* = 745) in the first phase of the study, and the ones who participated in the original cohort (*n* = 865). Comparison of the characteristics between the mothers included in the cohort and those who did not conclude the study showed no significant differences.

Table 1 presents the characteristics of the women included in the study. Considering that there is no clear cut-off point for the scores of the PSS, they are presented in tertiles. High scores of the GHQ, SAI and TAI in the three trimesters of pregnancy and 5–8 years after delivery varied from 23.3% to 58.4%.

**Table 1.** Characteristics of the women included in the study (n = 409)

	*N*	%	Mean (s.d.)
Age (years)			30.7 (6.06)
Education (years)^a^			7.7 (3.1)
*Per capita* income (MBW)^b^			0.92 (0.67)
Family size (number of persons)			4.3 (1.4)
Number of children			1.8 (0.97)
1	209	51.1	
2	116	28.4	
3	61	14.9	
⩾4	23	5.6	
Work			
Yes	114	27.9	
No	295	72.1	
Marital status			
Married/with partner	313	76.5	
Others	96	23.5	
BMI (weight/height^2^)			27.3 (4.1)
18.5–24.9 (normal)	124	30.3	
25.0–29.9 (overweight)	201	49.1	
30.0–39.9 (obese I and II)	84	20.6	
1st trimester			
PSS			22.7 (6.6)
7–20	138	33.6	
21–25	125	30.4	
26–43	147	36.0	
GHQ			2.8 (3.1)
⩽3	273	66.7	
⩾4	136	33.3	
SAI			37.3 (8.8)
<40	282	68.9	
⩾40	127	31.1	
TAI			41.8 (10.6)
<40	199	48.7	
⩾40	210	51.3	
2nd trimester			
PSS			22.8 (6.9)
4–19	132	32.3	
20–25	138	33.6	
26–46	140	34.1	
GHQ			2.3 (2.6)
⩽3	303	74.1	
⩾4	106	25.9	
SAI			35.9 (7.9)
<40	314	76.7	
⩾40	95	23.3	
TAI			40 (9.9)
<40	227	55.6	
⩾40	182	44.4	
3rd trimester			
PSS			22.1 (7.2)
2–19	147	35.8	
20–25	129	31.6	
26–53	134	32.6	
GHQ			2.5 (2.7)
⩽3	304	74.3	
⩾4	105	25.7	
SAI			36.8 (8.4)
<40	302	73.8	
⩾40	107	26.2	
TAI			39.5 (9.8)
<40	224	54.8	
⩾40	185	45.2	
5–8 years post-partum			
PSS			38.1 (7.4)
15–35	143	34.8	
36–41	136	33.3	
42–55	131	31.9	
GHQ			3.2 (3.2)
⩽3	252	61.6	
⩾4	157	38.4	
SAI			40.2 (10.1)
<40	228	55.7	
⩾40	181	44.3	
TAI			43.7 (10.8)
<40	170	41.6	
⩾40	239	58.4	

a *n* = 406.

b Minimum Brazilian Wage – MBW (1 MBW = R$350.00 = approx. US$77).

PSS, Perceived Stress Scale; GHQ, General Health Questionnaire; STAI, State-Trait Anxiety Inventories (SAI, State Anxiety Inventory; TAI-Trait Anxiety Inventory).

Table 2 shows four linear regression models considering scores of the PSS, GHQ, SAI and TAI 5–8 years after delivery as outcomes, and scores of the PSS, GHQ, SAI e TAI in the three trimesters of pregnancy as independent variables, controlling for confounders. Scores of the PSS 5–8 years after delivery were positively associated with scores of the PSS in the 1st and 3rd trimesters of pregnancy, and inversely associated with maternal age and *per capita* income (adj. *R*^2^ = 0.21). Scores of the GHQ 5–8 years after delivery were positively associated with scores of the GHQ in the three trimesters of pregnancy, and inversely associated with maternal age and *per capita* income (adj. *R*^2^ = 0.18). Scores of the SAI 5–8 years after delivery were positively associated with scores of the SAI in the 2nd and 3rd trimesters of pregnancy, and inversely associated with *per capita* income (adj. *R*^2^ = 0.15). Scores of the TAI 5–8 years after delivery were positively associated with scores of the TAI in the 1st and 2nd trimesters of pregnancy, and inversely associated with maternal age and *per capita* income (adj. *R*^2^ = 0.37).

**Table 2.** Linear regression models considering scores of the PSS, GHQ, SAI and TAI 5–8 years after delivery as outcomes

Outcomes	Coefficient	s.e.	*T*	95% CI		*p*
PSS (5–8 years after delivery)						
PSS (1st trimester)	0.20	0.08	2.54	0.05	0.36	0.01
PSS (2nd trimester)	0.13	0.09	1.50	−0.04	0.31	0.14
PSS (3rd trimester)	0.19	0.08	2.35	0.03	0.35	0.02
Maternal age	−0.15	0.07	−2.24	−0.29	−0.02	0.03
*Per capita* income	−0.67	0.23	−2.86	−1.13	−0.21	0.005
*R*^2^ = 0.23; Adj. *R*^2^ = 0.21						
GHQ (5–8 years after delivery)						
GHQ (1st trimester)	0.27	0.06	4.14	0.14	0.39	<0.001
GHQ (2nd trimester)	0.12	0.07	1.67	−0.02	0.27	0.09
GHQ (3rd trimester)	0.14	0.07	2.10	0.9 × 10^−2^	0.28	0.04
Maternal age	−0.07	0.03	−2.78	−0.12	−0.02	0.006
*Per capita* income	−0.24	0.09	−2.55	−0.42	−0.05	0.01
*R*^2^ = 0.19; Adj. *R*^2^ = 0.18						
SAI (5–8 years after delivery)						
SAI (1st trimester)	0.10	0.06	1.59	−0.02	0.23	0.11
SAI (2nd trimester)	0.16	0.08	2.10	0.01	0.31	0.04
SAI (3rd trimester)	0.28	0.07	4.18	0.15	0.42	<0.001
*Per capita* income	−0.84	0.29	−2.92	−1.41	−0.27	0.004
*R*^2^ = 0.16; Adj. *R*^2^ = 0.15						
TAI (5–8 years after delivery)						
TAI (1st trimester)	0.29	0.06	5.00	0.18	0.41	<0.001
TAI (2nd trimester)	0.25	0.07	3.62	0.12	0.39	<0.001
TAI (3rd trimester)	0.12	0.08	1.53	−0.03	0.26	0.13
Maternal age	−0.15	0.07	−2.10	−0.29	−0.9 × 10^−2^	0.04
*Per capita* income	−0.90	0.27	−3.34	−1.43	−0.37	0.001
*R*^2^ = 0.38; Adj. *R*^2^ = 0.37						

PSS, Perceived Stress Scale; GHQ, General Health Questionnaire; SAI, State Anxiety Inventory; TAI-Trait Anxiety Inventory.

Table 3 shows four linear regression models considering scores of the PSS, GHQ, SAI and TAI in the 3rd trimester of pregnancy as outcomes, and scores of the PSS, GHQ, SAI e TAI in the 1st and 2nd trimesters of pregnancy as independent variables, controlling for confounders. Scores of the PSS, GHQ, SAI and TAI in the 3rd trimester of pregnancy were positively associated with scores of the PSS, GHQ, SAI and TAI in the 1st and 2nd trimesters of pregnancy (adj. *R*^2^ varied from 0.31 to 0.65).

**Table 3.** Linear regression models considering scores of the PSS, GHQ, SAI and TAI in the 3rd trimester of pregnancy as outcomes

Outcomes	Coefficient	s.e.	*T*	95% CI		*p*
PSS (3rd trimester)						
PSS (1st trimester)	0.27	0.06	4.47	0.15	0.39	<0.001
PSS (2nd trimester)	0.54	0.06	8.95	0.42	0.66	<0.001
*R*^2^ = 0.47; Adj. *R*^2^ = 0.47						
GHQ (3rd trimester)						
GHQ (1st trimester)	0.20	0.05	4.38	0.11	0.29	<0.001
GHQ (2nd trimester)	0.49	0.05	10.17	0.39	0.59	<0.001
*R*^2^ = 0.35; Adj. *R*^2^ = 0.35						
SAI (3rd trimester)						
SAI (1st trimester)	0.23	0.05	4.97	0.14	0.33	<0.001
SAI (2nd trimester)	0.44	0.05	8.31	0.33	0.54	<0.001
*R*^2^ = 0.32; Adj. *R*^2^ = 0.31						
TAI (3rd trimester)						
TAI (1st trimester)	0.31	0.36	8.72	0.24	0.38	<0.001
TAI (2nd trimester)	0.56	0.04	14.54	0.48	0.63	<0.001
*R*^2^ = 0.65; Adj. *R*^2^ = 0.65						

PSS, Perceived Stress Scale; GHQ, General Health Questionnaire; SAI, State Anxiety Inventory; TAI-Trait Anxiety Inventory.

## Discussion

According to the results of this study, there were statistically significant associations between mental disorders (assessed by the PSS, GHQ and STAI scores) in the three trimesters of pregnancy, lower age and *per capita* income and mental disorders 5–8 years after delivery. The other socioeconomic and demographic factors investigated did not show statistically significant results. BMI, an indicator of the nutritional status of the women, was not associated with mental disorders; though Nagl *et al.* (2015) and Molyneaux *et al.* (2016) had referred that obese pregnant women might constitute a group vulnerable for anxiety and depression, respectively.

Apparently, women have a higher risk of mental disorders in the postpartum period if they have shown these disorders before (Bilszta *et al.* 2008; Witt *et al.* 2011; Kirkan *et al.* 2015; Patton *et al.* 2015) or during pregnancy (Onoye *et al.* 2013; Yazici *et al.* 2015). Bilszla *et al.* (2008) observed an association between history of psychopathology in the antenatal period and depression at 6–8 weeks postnatally in Australian women. Patton *et al.* (2015) observed that perinatal depressive symptoms were mostly preceded by mental health problems that began before pregnancy, in adolescence or young adulthood. Onoye *et al.* (2013) confirmed the associations between stress, depression and anxiety in the three trimesters of pregnancy and in early (<6 weeks after delivery) and late (⩾6 weeks after delivery) postpartum in women from Hawaii. Kirkan *et al.* (2015) and Yazici *et al.* (2015) reported that a previous mental disorder or an untreated depressive disorder in the 1st trimester of pregnancy was an important predictor of depression in the sixth postpartum week.

Comparing the mean scores of the women in the highest PSS tertile with the mean scores of the whole population, and the mean scores of the populations studied by Cohen *et al*. (1983), we concluded that the women included in our study had perceived stress in pregnancy and 5–8 years after delivery. Interestingly, PSS mean scores increased significantly (*t* test, *P* < 0.001; data not shown) 5–8 years after delivery compared with the mean scores in pregnancy, while the GHQ, SAI and TAI scores maintained the mean values in the four different periods investigated, indicating higher levels of perceived stress 5–8 years after delivery than in pregnancy. Schmied *et al.* (2013) observed that women's mood appears to be better in the first year after birth, when compared with a specific moment in pregnancy and 5 years later. The proportion of women reporting depressive symptoms in the first year postpartum was between 10% and 20% and this remained stable over 25 years. However, the studies included in the metanalysis carried out by Schmied *et al.* (2013) did not assess mental health in the three trimesters of pregnancy. Agrati *et al.* (2015) found that anxiety followed a U-shaped pattern from pregnancy to 2 years postpartum, which was modified by early life experience of the women. Greater early adversity was associated with higher anxiety in pregnancy, followed by a marked decrease once the baby was born, and a subsequent increase during the later postpartum period.

Similar to other studies in the literature, lower age (Bottino *et al.* 2012; Räisänen *et al.* 2014; Siegel & Brandon, 2014) and inferior *per capita* income (Abdollahi *et al.* 2014; Hein *et al.* 2014; Alfayumi-Zeadna *et al.* 2015) were associated with mental disorders in the postpartum period. According to Bottino *et al.* (2012), maternal age was significantly associated with PPD. For each additional year, a reduction of 4% in the chance of developing PPD was anticipated, effect that was not modified by confounders. Räisänen *et al.* (2014) referred that one of the risk profiles of major depression included adolescence or advanced maternal age. Primarily focusing upon depressive symptoms, findings from a large literature review showed that rates of depression in pregnant and postpartum adolescents are comparable with non-pregnant adolescents, but higher than those reported among pregnant adults (Siegel & Brandon, 2014). In fact, Aras *et al.* (2013) showed that early maternal age is one of the risk factors for depressive disorders even in non-perinatal reproductive age.

Abdollahi *et al.* (2014) identified low household income as the predictive factor with the highest risk (OR 3.57, 95% CI 1.49–8.5) for PPD. Hein *et al.* (2014) concluded that socioeconomic factors, including monthly income, define subgroups that have different depression scores during and after pregnancy. Alfayumi-Zeadna *et al.* (2015), using a validated Arabic translation of the ‘Edinburgh Postnatal Depression Scale’ found that low income was one of the socio-demographic factors associated with PPD, among Arab-Bedouin women.

A limitation of this study was the lack of assessment of mental disorders in the immediate post-partum period. Even though mental disorders had not been investigated in the immediate postpartum period, it was probably a problem for those pregnant women considering the associations of the PSS, GHQ and STAI scores between the 3rd and 1st and 2nd trimesters of pregnancy. Witt *et al.* (2011) referred that poor pre-pregnancy mental health and poor antepartum mental health, both independently, increased the risk of postpartum mental health problems in North American women. However, the authors did not use specific questionnaires to assess mental disorders. They used self-reports and symptoms of mental health conditions or global mental health ratings of ‘fair’ or ‘poor’.

## Conclusion

Mental disorders in the three trimesters of pregnancy, lower age and *per capita* income are associated with mental disorders 5–8 years after delivery.

Referral of young, low-income pregnant women with mental disorders, particularly anxiety and depression, should be encouraged to psychological or psychiatric treatment. Finally, we reinforce the urgency to integrate mental health screening into routine primary care for pregnant and postpartum women.
